# Exploring the relationship between vitamin B12, methylmalonic acid levels and all-cause mortality in heart failure populations: insights from the NHANES database

**DOI:** 10.3389/fnut.2025.1597305

**Published:** 2025-06-25

**Authors:** Wei Yu, Yao Wang, Yulu Zhou, Danyu Wu

**Affiliations:** ^1^Department of Surgical Oncology, Sir Run Run Shaw Hospital, Zhejiang University School of Medicine, Hangzhou, China; ^2^Department of Cardiology, Sir Run Run Shaw Hospital, Zhejiang University School of Medicine, Hangzhou, China; ^3^Zhejiang Key Laboratory of Cardiovascular Intervention and Precision Medicine, Hangzhou, China; ^4^Engineering Research Center for Cardiovascular Innovative Devices of Zhejiang Province, Hangzhou, China

**Keywords:** vitamin B12, methylmalonic acid, heart failure, mortality, NHANES

## Abstract

**Background:**

A functional deficiency in vitamin B12 is a prevalent condition among heart failure (HF) patients. Despite being a specific and sensitive marker for this deficiency, the study of methylmalonic acid (MMA) in the context of HF has been infrequent.

**Methods:**

Seven hundred and forty-seven individuals with HF were incorporated in this cross-sectional study who participated in the National Health and Nutrition Examination Survey (NHANES) from two periods, 1999 to 2004, and 2011 to 2014. Hazard ratios (HRs) and 95% confidence intervals (CIs) of the risk of all-cause mortality were estimated using weighted multivariable Cox proportional hazard models. The non-linear association between MMA levels and all-cause mortality was investigated using restricted cubic spline (RCS) analyses.

**Results:**

Among 747 HF participants, 481 (57.3%) deaths were recorded during a follow-up period of 7.9 years. Elevated serum MMA levels were significantly associated with an increased risk of all-cause mortality (Tertile 3 compared with Tertile 1: adjusted HR: 1.52; 95% CI: 1.09, 2.13; *p* = 0.01), demonstrating a dose–response pattern (26% increased mortality risk per unit increase in lnMMA). B12 intake from diet was not significantly associated with mortality risk (*p* = 0.81). Although a minor statistically significant association was observed in serum B12 levels and mortality (*p* = 0.045), it disappeared after multivariate regression analysis. Moreover, the correlation between MMA and mortality risk was more prominent in HF populations with poorer health status, such as advanced age, current smokers, hypertension, diabetes, overweight, or low estimated glomerular filtration rate (eGFR).

**Conclusion:**

Our study indicated that a higher MMA level is associated with an increased all-cause mortality risk in HF populations, particularly in those aged 65 and above, current smokers, those with hypertension, diabetes, overweight, insufficient physical activity, or lower eGFR (<60 mL/min/1.73 m^2^).

## 1 Introduction

Heart failure (HF) represents a severe manifestation or an advanced stage of a range of cardiovascular diseases (CVDs), carrying substantial social and economic implications. The incidence and mortality rates of HF continue to rise over time and are anticipated to remain high in the foreseeable future (1). Despite the multitude of pathophysiological mechanisms involved, targeting specific metabolic derangements, such as visceral adiposity dysfunction and insulin resistance (2), is expected to provide therapeutic and prognostic indicators. In heart failure, there can be disruptions or deficiencies in various micronutrients, including vitamins and minerals (3–5). In particular, deficiencies in several vitamins, such as vitamin D, B1, B2, B6, and B12, are commonly observed (6–9).

Vitamin B12, also known as cobalamin, plays a crucial role in neurological function, haemopoiesis, and DNA synthesis. It acts as a necessary cofactor in two key biochemical reactions: the transformation of methylmalonic acid (MMA) into succinyl-CoA, and the transformation of homocysteine (Hcys) into methionine. Up to 5–10% of patients with heart failure have been found to have a deficiency in vitamin B12, typically identified by reduced serum total B12 levels (10). However, a B12 deficiency can occur even when B12 levels appear normal and it may result in neurological symptoms without any hematologic abnormalities (11).

In case of functional B12 deficiency, the levels of serum MMA and/or Hcys can be found to be unusually high. This elevation is a result of decreased enzymatic transformation. These are sensitive biochemical markers for diagnosing B12 deficiency even when serum B12 concentrations fall within the normal or marginally low ranges (12). This is particularly important in HF patients, where systemic inflammation, oxidative stress, and altered renal function can interfere with B12 metabolism and transport, leading to discrepancies between serum B12 levels and actual metabolic activity. Hcys is recognized as an independent risk factor for cardiovascular diseases (13) but can also rise in conditions of folate, thiamine, or vitamin B6 deficiency. Elevated MMA levels, by directly reflecting impaired conversion to succinyl-CoA, serve as a more specific and sensitive indicator of functional B12 deficiency and its downstream metabolic consequences (14–16).

Studies have observed a significant increase in serum MMA levels in patients with acute myocardial infarction or acute heart failure in comparison to healthy individuals (17, 18). Severe cardiomyopathy has also been detected in patients diagnosed with methylmalonic acidemia, a metabolic disorder inherited in an autosomal recessive manner (19). Furthermore, a robust correlation has been found between MMA levels and both all-cause mortality and cardiovascular mortality in the general population, particularly among individuals with functional vitamin B12 deficiency (20, 21).

Despite these findings, only a handful of studies have delved into the association of MMA levels with HF mortality. Therefore, we carried out a population-based study to investigate whether elevated MMA levels are associated with a heightened all-caused mortality in HF individuals.

## 2 Materials and methods

### 2.1 Study population

The National Health and Nutrition Examination Survey (NHANES), an ongoing cross-sectional study conducted in every two-year cycle, screens approximately 10,000 participants nationwide on the basis of multistage and stratified random sampling. The survey collects information on a variety of factors including demographics, socioeconomic factors, diet and health-related issues through home interviews. Physical examinations and laboratory tests are performed in Mobile Examination Centers (MEC). The National Center for Health Statistics (NCHS) Research Ethics Review Board has approved the research protocols, and informed consent has been obtained from all participants. Detailed information can be found on the NHANES website^1^.

This study incorporated data from a total of 51,057 participants across five NHANES cycles (1999–2000, 2001–2002, 2003–2004, 2011–2012 and 2013–2014). Data on MMA were not accessible for the NHANES cycles 2005–2010 and 2015–2020. Participants without MMA data (*n* = 12,685) were excluded. 38,372 participants with MMA data were screened using the MCQ160B questionnaire (Ever told had congestive heart failure?) and selected if their answer was “Yes” (*n* = 747).

**Figure 1:**
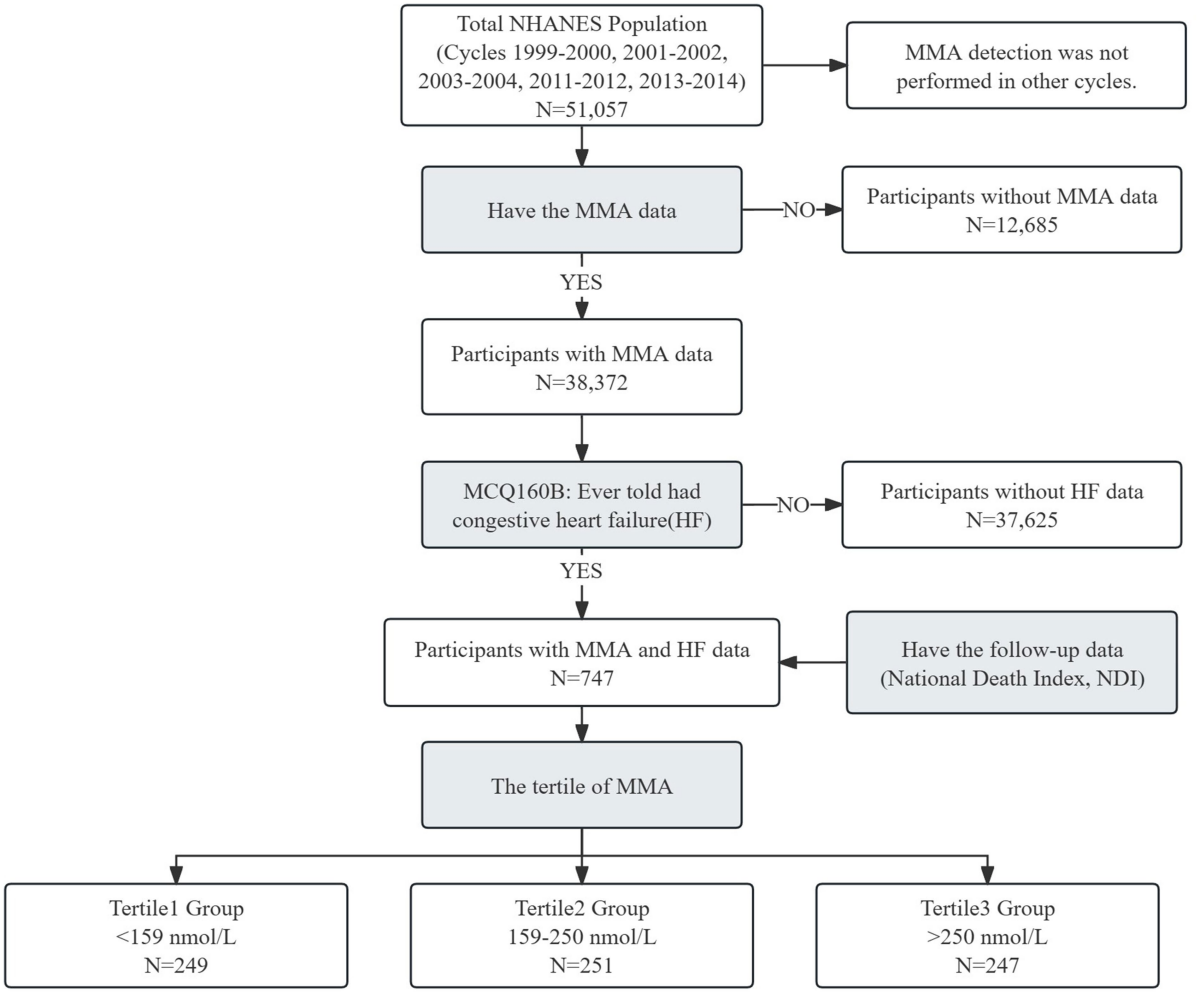
The flowchart of this study.

The study’s flowchart is depicted in Figure 1. Seven hundred and forty-seven participants with both MMA and HF data were enrolled and then divided into three groups according to the tertiles of MMA levels. The groups were defined as follows: Tertile 1 group, with MMA < 159 nmol/L (*n* = 249); Tertile 2 group, with 159 nmol/L ≤ MMA ≤ 250 nmol/L (*n* = 251); and Tertile 3 group, with MMA > 250 nmol/L (*n* = 247).

### 2.2 Measurement of MMA and vitamin B12

In the NHANES cycles from 1999 to 2004, MMA was extracted from plasma or serum samples (275 μL). This extraction process was conducted using a commercially available strong anion exchange resin. The MMA level was measured by gas chromatography–mass spectrometry (GC/MS). In the NHANES 2011–2014, MMA was extracted from serum (75 μL) via liquid–liquid extraction. The MMA level was measured by liquid chromatography–tandem mass spectrometry (LC–MS/MS). In this study, the units used to quantify MMA levels were in nanomoles per liter (nmol/L).

Vitamin B12 was measured by using the Bio-Rad Laboratories “Quantaphase II Folate/vitamin B12” Radioassay Kit (Bio-Rad Laboratories, 1993) in the NHANES from 1999 to 2004 and using the fully automated electrochemiluminescence immunoassay “ECLIA” in National Center for Environmental Health in the NHANES from 2011 to 2014. In this study, the units used to quantify vitamin B12 levels were in picogram per milliliter (pg/mL). Serum B12 grouping were determined using tertile-based classification as follows: Tertile 1 (<387 pg./mL), Tertile 2 (387–606 pg./mL), and Tertile 3 (>606 pg./mL).

### 2.3 Ascertainment of mortality

The primary outcome of this study was all-cause mortality among the heart failure population. Follow-up data were collected from the public-use database in the National Death Index (NDI) up until 2019. All-cause mortality was defined as death due to any cause. Participants who did not have a recorded death during the follow-up period were assumed to be alive.

### 2.4 Measurement of covariates

Data concerning various demographic and lifestyle factors were collected from the baseline standardized questionnaires. These factors included age, gender (male and female), race (other Hispanic, non-Hispanic White, non-Hispanic Black, and others), education level (less than high school, high school, and college or higher), Poverty income ratio (PIR), smoking status (never, former, and current), drinking status (yes or no), history of hypertension (yes or no), history of diabetes (yes or no), and NYHA class (I/II or III/IV). NYHA classification was performed using the CDQ010 questionnaire (had shortness of breath either when hurrying on the level or walking up a slight hill?). Participants with the answer “Yes” were classified as NYHA III/IV, while those who answered “no” were classified as NYHA I/II. Physical activity was categorized as sufficient if it was more than 600 min-Mets, and insufficient otherwise (22). The Body Mass Index (BMI) was computed by dividing the weight of the individuals (in kilograms) by the square of their height (in meters squared). Based on laboratory data, eGFR was calculated using the Chronic Kidney Disease Epidemiology Collaboration (CKD-EPI) equation. B12 intake from diet was collected using a 24-h dietary recall. This was done on 1 day during the NHANES cycles 1999–2000, and 2001-2002, and on two consecutive days from the year 2003 onward. B12 intake grouping were determined using tertile-based classification as follows: Tertile 1 (<2.55 μg/d), Tertile 2 (2.55–4.54 μg/d), and Tertile 3 (>4.54 μg/d).

### 2.5 Statistical analysis

Data were presented as a count (%) for categorical variables and as a mean ± standard deviation for continuous variables. All analyses used sampling weights to extrapolate results to the civilian non-institutionalized resident population of the United States. Baseline characteristics were compared using the *χ*^2^ test or Kruskal–Wallis test. Univariate Cox regression was conducted for MMA, serum B12, and B12 intake from diet, respectively, to predict overall mortality. The event-free survival was gauged by generating Kaplan–Meier plots. We utilized four adjustment models of multivariate Cox proportional hazard to analyze the relationship between MMA and overall mortality. Model 1 was adjusted for age, gender, and races. Model 2 was adjusted for age, gender, races, smoke, hypertension, physical activity, and eGFR. Model 3 was additionally adjusted for B12 intake from diet based on model 2, while model 4 adjusted for serum B12 based on model 2. The difference between groups was evaluated using the log-rank test. Any missing data were omitted from the analysis. Sensitivity analysis using the Random Forest imputation model was performed to evaluate the potential impact of missing data of serum B12. All statistical analyses and graph plotting were performed using R statistical software, version 4.3.2. A *p*-value less than 0.05 was deemed statistically significant. All the raw codes and data used in this study can be found in the Supplementary material, labeled as Rawcode.zip.

## 3 Results

### 3.1 Baseline characteristics

During the identified study period, a total of 747 HF populations with MMA data were enrolled from NHANES. Table 1 presents the descriptive baseline characteristics of the study population, which is divided into three groups.

**Table 1:** Baseline characteristics of the heart failure participants by the tertile of MMA levels.

Characteristic		Participants, *N*				*p* ^*^
			MMA Levels, nmol/L			
		Total	Tertile 1 (<159)	Tertile 2 (159–250)	Tertile 3 (>250)	
Participants, *N* (%)		747 (100.0)	249 (100.0)	251 (100.0)	247 (100.0)	
Age, years		65.69 ± 14.28	60.07 ± 13.79	67.65 ± 13.65	69.84 ± 13.53	<0.01
Sex, *N* (%)	Female	359 (51.3)	118 (47.6)	115 (47.0)	126 (59.8)	0.05
	Male	388 (48.7)	131 (52.4)	136 (53.0)	121 (40.2)	
Race, *N* (%)	Other Hispanic	137 (9.0)	68 (13.7)	34 (5.8)	35 (7.3)	0.05
	Non-Hispanic White	437 (76.0)	117 (68.8)	152 (78.8)	168 (81.1)	
	Non-Hispanic Black	148 (11.3)	56 (12.3)	56 (12.2)	36 (9.2)	
	Other Race	25 (3.7)	8 (5.2)	9 (3.2)	8 (2.4)	
Education level, *N* (%)	Less than high school	331 (35.9)	112 (36.4)	108 (33.3)	111 (38.2)	0.75
	High school diploma	163 (25.3)	54 (22.5)	56 (27.2)	53 (26.3)	
	More than high school	252 (38.8)	83 (41.1)	86 (39.6)	83 (35.4)	
PIR, ratio		2.22 ± 1.45	2.20 ± 1.50	2.35 ± 1.46	2.12 ± 1.38	0.42
Smoking status, *N* (%)	Never smoking	309 (37.6)	99 (33.8)	106 (38.4)	104 (41.0)	0.03
	Former smoker	306 (40.8)	87 (36.1)	116 (46.1)	103 (40.3)	
	Current smoker	132 (21.6)	63 (30.1)	29 (15.5)	40 (18.7)	
Drinking, *N* (%)	Yes	360 (64.9)	128 (67.7)	118 (68.5)	114 (57.9)	0.18
	No	208 (35.1)	66 (32.3)	67 (31.5)	75 (42.1)	
Hypertension, *N* (%)	Yes	563 (72.2)	179 (65.6)	190 (72.0)	194 (79.7)	0.03
	No	183 (27.8)	69 (34.4)	61 (28.0)	53 (20.3)	
Diabetes, *N* (%)	Yes	291 (35.2)	90 (30.4)	100 (38.0)	101 (37.5)	0.28
	No	456 (64.8)	159 (69.6)	151 (62.0)	146 (62.5)	
Physical activity, *N* (%)	Insufficient	437 (64.1)	136 (56.8)	156 (67.9)	145 (68.8)	0.1
	Sufficient	196 (35.9)	83 (43.2)	59 (32.1)	54 (31.2)	
BMI, kg/m^2		31.37 ± 7.93	31.91 ± 7.57	31.85 ± 7.90	30.26 ± 8.30	0.16
eGFR, mL/min/1.73 m^2		73.64 ± 34.38	91.59 ± 35.69	71.99 ± 26.03	55.00 ± 30.08	<0.01
MMA, nmol/L		262.75 ± 245.83	121.66 ± 24.61	203.07 ± 27.07	479.24 ± 339.92	<0.01
B12 intake from diet, μg/d		4.32 ± 3.69	4.29 ± 2.84	4.63 ± 4.98	4.02 ± 2.79	0.35
Serum B12, pg./mL		576.73 ± 400.92	608.32 ± 354.42	641.71 ± 503.53	477.77 ± 309.33	<0.01
NYHA class	I/II	195 (25.8)	60 (22.4)	74 (30.8)	61 (24.4)	0.24
	III/IV	525 (74.2)	176 (77.6)	170 (69.2)	179 (75.6)	
Mortality, *N* (%)	Yes	481 (57.3)	124 (41.3)	162 (58.3)	195 (73.6)	<0.01
	No	266 (42.7)	125 (58.7)	89 (41.7)	52 (26.4)	
Follow-up time, months		95.44 ± 63.97	116.29 ± 62.80	92.66 ± 64.61	75.41 ± 57.57	<0.01

MMA, methylmalonic acid; PIR, family poverty-to-income ratio; BMI, body mass index; eGFR, estimated glomerular filtration rate; B12, cobalamin (vitamin B12); NYHA, New York Heart Association.

Variables are presented as the unweighted number with the weighted proportion (%) or mean ± SE (stand error).

*p-values were calculated using the Wald and F test for the continuous variables, and the Rao–Scott chi-square test for the categorical variables.

On average, the participants were 65.69 ± 14.28 years old with an average PIR of 2.22 ± 1.45. 51.3% of the populations were female, and 76.0% were non-Hispanic White. Regarding education, 38.8% had more than a high school education. In terms of lifestyle factors, 62.4% had a history of smoking (with 40.8% being former smokers and 21.6% being current smokers), 64.9% consumed alcohol, and 64.1% had insufficient physical activity. 72.2% of the populations had hypertension, while 64.8% did not have diabetes. The average BMI was 31.37 ± 7.93. Focusing on the severity of HF, 74.2% of the participants were classified as NYHA Class III/IV. The distribution of NYHA classification across the tertiles showed no significant differences, with Tertile 1 at 77.6%, Tertile 2 at 69.2%, and Tertile 3 at 75.6% (*p* = 0.24).

The average MMA levels for the three groups, which were divided according to tertiles, were 121.66 ± 24.61 nmol/L, 203.07 ± 27.07 nmol/L, and 479.24 ± 339.92 nmol/L. Participants with elevated MMA levels tended to be older, non-Hispanic White, non-smokers, and hypertension patients, and to have lower eGFR. Interestingly, while the average B12 intake from diet did not significantly differ between groups, the serum B12 level was highest in Tertle 2 group with a significant difference (641.71 ± 503.53 pg./mL versus 608.32 ± 354.42 pg./mL in Tertle 1 and 477.77 ± 309.33 pg./mL in Tertle 3, *p* < 0.01).

### 3.2 Main outcomes

**Figure 2:**
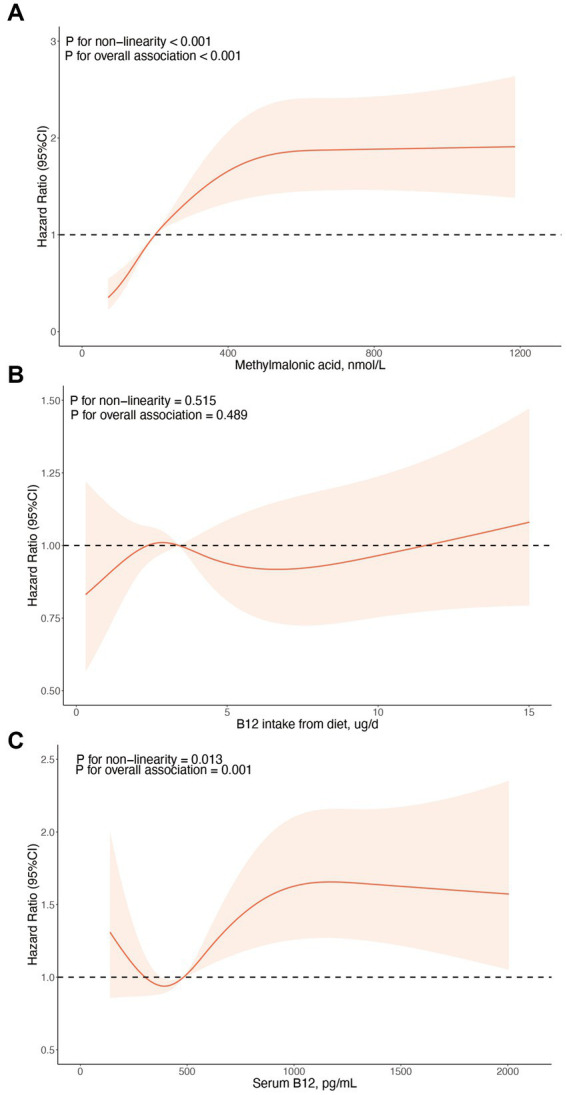
Association of the MMA, B12 intake from diet, and serum B12 with all-cause mortality among participants with heart failure, evaluated using restricted cubic splines. **(A)** Association of the MMA with all-cause mortality. **(B)** Association of the B12 intake from diet with all-cause mortality. **(C)** Association of the serum B12 with all-cause mortality. The results are presented using solid red lines for the hazard ratios, light red shadow (indicating the 95% confidence interval) for the confidence intervals and black dashed lines for hazard ratios for reference lines of 1.0. *p*-values for non-linearity and overall association are also presented.

**Figure 3:**
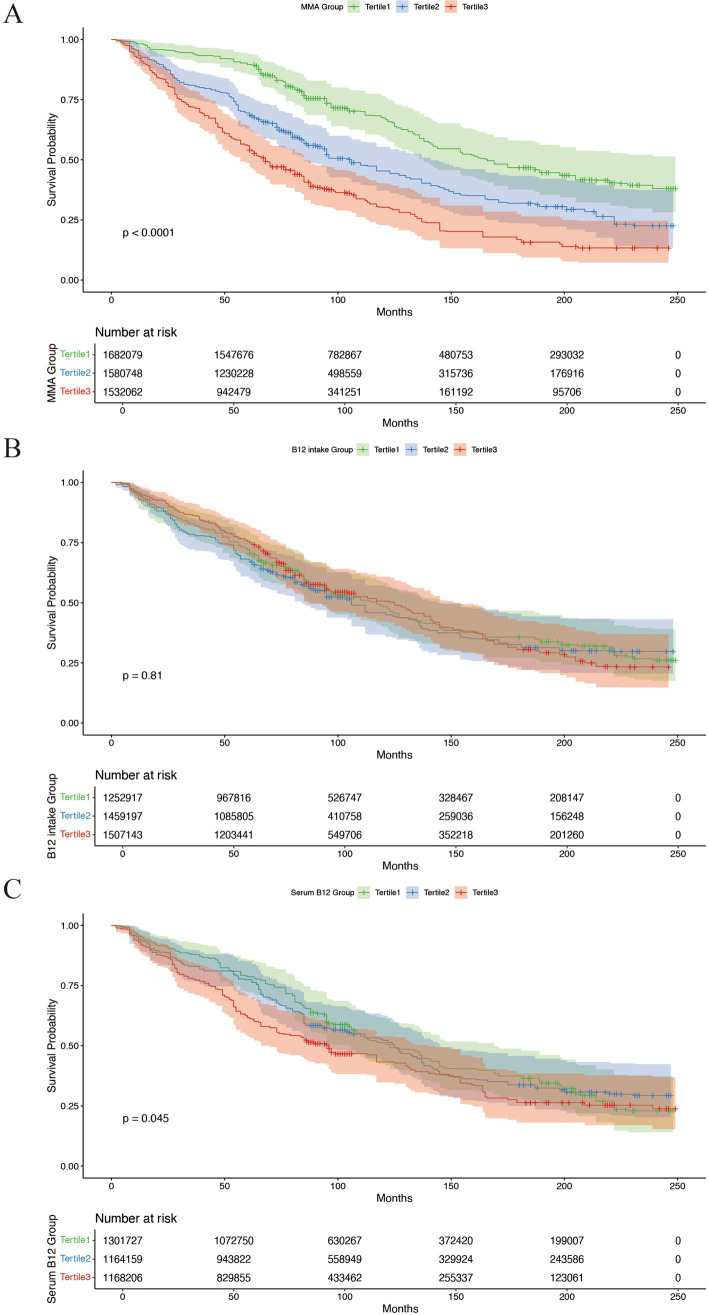
Accumulative survival probability by MMA, B12 intake from diet, and serum B12 strata. **(A)** Accumulative survival probability by baseline MMA strata. **(B)** Accumulative survival probability by baseline B12 intake from diet strata. **(C)** Accumulative survival probability by baseline serum vitamin B12 strata. Weighted Kaplan–Meier plots between all-cause mortality and MMA, B12 intake from diet, and serum vitamin B12. The event-free survival was gauged by generating Kaplan–Meier plots. *p*-value was estimated by the log-rank test.

During the average follow-up time of 95.44 months, 481 (57.3%) deaths from any causes was recorded. Restricted cubic splines revealed a non-linear association between MMA levels and all-cause mortality (*p* < 0.001, Figure 2). Consistently, weighted Kaplan–Meier plots also indicated a significant increase in all-cause mortality risk in the higher MMA tertile (*p* < 0.0001, Figure 3). Concurrently, a higher MMA level was associated with a shorter follow-up period (*p* < 0.01, Table 1), which could be related to the increase in mortality. In contrast, B12 intake from diet was not significantly associated with all-cause mortality in the HF populations, as indicated by the spline fitting and weighted Kaplan–Meier plots (Figures 2, 3).

We conducted weighted multivariable Cox regression analyses to assess the mortality risk related to MMA (Table 2). A significant correlation was observed between the baseline serum MMA level and an elevated all-cause mortality risk. The multivariable-adjusted HRs and 95% CIs in Tertile 2 and Tertile 3 were HR: 1.24, 95% CIs: 0.89–1.74 and HR: 1.52, 95% CIs: 1.09–2.13, respectively, compared with Tertile 1 (*p* = 0.01), as per model 2. When MMA was treated as a continuous variable, a 26% increase in the mortality risk per unit increase in lnMMA was still observed. Given the close relationship between vitamin B12 metabolism and MMA, we separately included B12 intake from diet and serum B12 levels as confounding factors. B12 intake from diet was found to have no significant effect on the results (as per model 3, with HR: 1.37, 95% CIs: 1.11–1.68). However, the significant association between MMA and all-cause mortality in HF populations disappeared when serum B12 levels were included for adjustment (as per model 4, with HR: 1.24, 95% CIs: 0.97–1.59, *p* = 0.08).

**Table 2:** Association of the MMA levels with all-cause mortality among participants with heart failure.

All-cause mortality	Participants, HR (95% CIs)					
			MMA Levels, nmol/L			
	Ln MMA^*^	*P*-value	Tertile 1 (<159)	Tertile 2 (159–250)	Tertile 3 (>250)	*p* trend^a^
Participants, *N* (%)	747 (100.0)		249 (100.0)	251 (100.0)	247 (100.0)	
Deaths/person-yrs	481/2825^&^		124/1009	162/911	195/905	
Crude	1.92 (1.56–2.35)^#^	<0.01	1(ref.)	1.80 (1.30–2.50)	2.83 (2.05–3.91)	<0.01
Model 1	1.56 (1.31–1.87)	<0.01	1(ref.)	1.37 (1.03–1.82)	1.98 (1.49–2.66)	<0.01
Model 2	1.26 (1.01–1.57)	0.04	1(ref.)	1.24 (0.89–1.74)	1.52 (1.09–2.13)	0.01
Model 3	1.37 (1.11–1.68)	<0.01	1(ref.)	1.28 (0.93–1.77)	1.66 (1.15–2.38)	0.02
Model 4	1.24 (0.97–1.59)	0.08	1(ref.)	1.20 (0.84–1.72)	1.33 (0.92–1.92)	0.12

^*^Hazard ratio per 1 unit increases of natural log-transformed MMA.

^&^Unweighted.

^#^Values are weighted hazard ratio (95% confidence interval).

^a^*p*-values for trend were assessed using the median level of each quartile MMA levels and modeling it as a continuous variable.

CI, confidence interval; HR, hazard ratio; and MMA, methylmalonic acid.

Model 1: adjusted for the Age (continuous, year), and Sex (male and female), and Race (Other Hispanic, non-Hispanic White, non-Hispanic Black, and other race).

Model 2: adjusted for the Age (continuous, year), and Sex (male and female), Race (Other Hispanic, non-Hispanic White, non-Hispanic Black, and other race), Smoking status (Never smoking, Former smoker, and Current smoker), Hypertension (Yes and No), Physical activity (Insufficient and Sufficient), and eGFR (continuous, mL/min/1.73 m^2).

Model 3: further adjustment for vitamin B12 intake from diet (continuous, ug/d) based on Model 2.

Model 4: further adjustment for serum vitamin B12 (continuous, pg/mL) based on Model 2.

### 3.3 Serum B12 and mortality

We observed a non-linear relationship between serum B12 levels and mortality in HF populations (*p* = 0.013, Figure 2). The mortality risk initially decreased and then increased as the serum B12 level rose, according to the restricted cubic spline. The association between serum B12 and all-cause mortality was nearly non-significant when examined by weighted Kaplan–Meier plots (*p* = 0.045). Moreover, compared to those with serum B12 levels <387 pg./mL (Tertile 1), the multivariable-adjusted HRs (95%) and CIs of all-cause mortality in participants with serum B12 levels of 387–606 pg./mL (Tertile 2) and >606 pg./mL (Tertile 3) were 1.37 (95% CI: 0.99, 1.89) and 1.38 (95% CI: 0.95, 1.98), respectively, with a *p*-value of 0.08, after adjusting for age, gender, races, smoke, hypertension, physical activity, and eGFR (as per model 2).

To address the exclusion of 167 participants due to missing B12 data, we performed sensitivity analyses using Random Forest model (Supplementary Table 1). The results was robust when analyzing serum B12 continuously, with the HR 1.17 (95% CI: 0.92–1.50, *p* = 0.21) in complete-case analysis and 1.24 (95% CI: 0.98–1.59, *p* = 0.08) after imputation. Nevertheless, the categorical analysis demonstrated significant changes: the HRs changed to 1.10 (95% CI: 0.82–1.51) for Tertile 2 and 1.54 (95% CI: 1.11–2.14) for Tertile 3, with a significant *p*-value <0.01, following imputation.

### 3.4 Stratified analyses for mortality risk associated with MMA

In the stratified analyses, the dose–response association between MMA and all-cause mortality was stratified by age, smoking status, hypertension, diabetes, physically active, BMI, eGFR, and serum B12 (all *p* ≥ 0.06 for interaction) (Table 3). Specifically, the mortality risk associated with MMA was higher in populations who were older than 65 years, current smokers, hypertensive, diabetic, overweight (with a BMI ≥ 30), physically inactive or had a lower eGFR (<60 mL/min/1.73 m^2^). Interestingly, the association between MMA and all-cause mortality is more pronounced in individuals with serum B12 levels ≥ 483.5 pg./mL (HR 2.00, 95% CI 1.31–3.07, *p* < 0.01), suggesting that MMA’s prognostic value is likely independent of B12 deficiency. These subgroup findings, while suggestive, require cautious interpretation due to the exploratory nature of the analyses and inherent limitations in subgroup sample sizes.

**Table 3:** Subgroup analyses for the HRs of the MMA levels and all-cause mortality among participants with heart failure.

Subgroup		Ln MMA	*p*-value	MMA levels (nmol/L), HR (95% CI)			*p*-value for interaction
				Tertile 1 (<159)	Tertile 2 (159–250)	Tertile 3 (>250)	
Age, years							0.11
	<65	1.17 (0.70–1.97)	0.55	1(ref.)	1.08 (0.53–2.20)	1.25 (0.55–2.87)	
	> = 65	1.33 (1.02–1.72)	0.03	1(ref.)	1.36 (0.92–2.02)	1.74 (1.17–2.58)	
Sex							0.56
	Female	1.14 (0.88–1.48)	0.32	1(ref.)	1.45 (0.87–2.41)	1.31 (0.84–2.03)	
	Male	1.39 (0.95–2.04)	0.09	1(ref.)	1.12 (0.71–1.75)	1.87 (1.13–3.10)	
Smoking status							0.31
	Never smoking	1.19 (0.92–1.54)	0.19	1(ref.)	1.79 (1.06–3.00)	1.73 (1.07–2.78)	
	Former smoker	1.43 (0.90–2.26)	0.13	1(ref.)	1.15 (0.66–2.00)	1.74 (0.96–3.16)	
	Current smoker	1.54 (0.96–2.44)	0.02	1(ref.)	0.96 (0.44–2.08)	1.34 (0.74–2.41)	
Drinking							0.24
	Yes	1.15 (0.81–1.62)	0.44	1(ref.)	1.03 (0.63–1.67)	1.24 (0.75–2.05)	
	No	1.29 (0.86–1.93)	0.21	1(ref.)	1.39 (0.71–2.74)	1.95 (1.00–3.80)	
Hypertension							0.2
	Yes	1.45 (1.10–1.91)	0.01	1(ref.)	1.53 (1.03–2.28)	1.81 (1.23–2.66)	
	No	0.92 (0.60–1.42)	0.71	1(ref.)	0.78 (0.42–1.47)	1.05 (0.49–2.23)	
Diabetes							0.98
	Yes	1.48 (0.99–2.20)	0.05	1(ref.)	1.74 (1.00–3.04)	2.38 (1.32–4.27)	
	No	1.10 (0.84–1.45)	0.5	1(ref.)	1.13 (0.75–1.70)	1.29 (0.86–1.93)	
Physical activity							0.76
	Insufficient	1.34 (1.04–1.73)	0.02	1(ref.)	1.22 (0.84–1.78)	1.67 (1.11–2.49)	
	Sufficient	1.13 (0.70–1.81)	0.62	1(ref.)	1.23 (0.63–2.40)	1.27 (0.68–2.35)	
BMI, kg/m^2							0.06
	<30	1.26 (0.94–1.67)	0.12	1(ref.)	1.37 (0.86–2.19)	1.28 (0.79–2.09)	
	> = 30	1.66 (1.07–2.60)	0.02	1(ref.)	1.40 (0.79–2.48)	2.08 (1.11–3.90)	
eGFR, mL/min/1.73 m^2							0.14
	<60	1.74 (1.34–2.27)	<0.01	1(ref.)	1.71 (0.90–3.26)	2.57 (1.43–4.62)	
	> = 60	1.14 (0.82–1.58)	0.43	1(ref.)	1.41 (0.89–2.24)	1.60 (0.96–2.68)	
B12 intake from diet, ug/d	<3.4	1.20 (0.86–1.68)	<0.05	1(ref.)	1.15 (0.71–1.85)	1.22 (0.73–2.04)	0.37
	> = 3.4	1.32 (0.91–1.91)	0.14	1(ref.)	1.30 (0.76–2.22)	2.15 (1.28–3.61)	
Serum B12, pg./mL	<483.5	0.98 (0.69–1.38)	0.89	1(ref.)	0.66 (0.39–1.13)	0.90 (0.55–1.46)	0.71
	> = 483.5	2.00 (1.31–3.07)	<0.01	1(ref.)	2.37 (1.41–4.00)	2.72 (1.53–4.86)	

HR, hazard ratio; MMA, methylmalonic acid; CI, confidence interval; BMI, body mass index; eGFR, estimated glomerular filtration rate; B12, cobalamin (vitamin B12).

## 4 Discussion

In this cross-sectional study involving 747 individuals from a nationally representative sample of the United States, we discovered that increasing MMA levels was significantly correlated with all-cause mortality in HF populations. This correlation persisted after adjusting for various risk factors, including demographics, race, smoking status, hypertension, physical activity, eGFR, and vitamin B12 intake from diet. The association between MMA and mortality also remained consistent after comprehensive stratified analyses, emphasizing the solidity of our findings. However, after further adjusting for serum B12 levels based on model 2, the association became non-significant (*p* = 0.08). Possible explanations for this outcome include: (1) Limited participants with both HF and MMA data reduced statistical power; (2) considerable missing data on serum B12 levels (167 missing out of 747 participants) further reduced power; (3) the relationship between vitamin B12 and mortality may be indirectly represented by MMA due to its role in MMA metabolism.

Vitamin B12, a cofactor for the enzyme methylmalonyl-CoA mutase, plays a crucial role when MMA is converted to succinyl-CoA. This conversion is vital for the normal metabolism of certain amino acids like valine, methionine, isoleucine, and threonine, as well as for the breakdown of odd-chain fatty acids. In the event of vitamin B12 deficiency, the activity of methylmalonyl-CoA mutase is hampered, resulting in a buildup of MMA within the body. This accumulation can cause an increase in MMA levels in both blood and urine.

According to the restricted cubic spline analysis of this cohort (Figure 2), the mortality risk first decreases and then increases as the serum B12 level rises. This pattern suggests that vitamin B12 is only beneficial within a certain range. A similar outcome was observed in another NHANES data-based study, which assessed the correlation between serum B12 concentration, vitamin B12 supplement intake and mortality from all causes, cardiovascular diseases, and cancer in the general population (23). This study revealed that lower serum B12 levels (<140 pmol/L) were linked to a minor elevation in both all-cause and cardiovascular mortality. Conversely, higher serum B12 levels (>700 pmol/L) were connected only with an increase in cardiovascular mortality. Notably, a higher intake of vitamin B12 supplement showed no correlation with increased mortality, suggesting its safety. The LURIC study (24) also revealed a U-shaped relationship between serum B12 levels and mortality. Researchers hypothesized that low B12 levels might contribute to mortality through elevated homocysteine levels, whereas high B12 levels could be associated with heightened inflammation. Additionally, a recent systematic review and dose–response meta-analysis (25) showed a linear positive correlation between high serum vitamin B12 levels (>600 pmol/L) and all-cause mortality risk, particularly pronounced in older adults. Collectively, these findings reveal a complex interplay between serum vitamin B12 and mortality. Current researches suggest that vitamin B12 deficiency increases mortality risk mainly through pathways such as a heightened risk of cardiovascular diseases (largely due to elevated Hcys), increased cardiac strain from anemia, impaired neurocognitive function (raising the likelihood of dementia), and greater susceptibility to frailty (26, 27). On the other hand, the mechanisms underlying the positive association between high B12 levels and increased mortality remain incompletely understood. However, several hypotheses have been suggested. Elevated serum B12 levels has been observed in various acute and chronic conditions, including liver failure, cirrhosis, hepatitis, interstitial nephropathy, chronic renal failure, inflammatory and infectious diseases, and malignancies (28–31). In these contexts, primary disease processes may lead to secondary increases in circulating B12, either through increased release or reduced clearance. Consequently, high serum B12 is often regarded as a consequence or biomarker of impaired liver function, organ damage or chronic diseases, rather than a direct cause of mortality.

It’s important to note that only about a quarter of circulating serum vitamin B12 is in an active form available for cells, achieved by binding to transcobalamin (32). Moreover, increased oxidative stress could lead to a functional deficiency of vitamin B12, a scenario that can occur even in the presence of high serum B12 levels (33). Therefore, measuring total serum vitamin B12 alone may not serve as sensitive marker for B12 deficiency. In cases of functional B12 deficiency, the metabolites MMA and/or Hcys may be abnormally elevated due to reduced enzymatic conversion (12). Generally, Hcy has low specificity as its levels can also elevate in patients with deficiencies of folate, thiamine or vitamin B6 (34). MMA is a more specific and sensitive functional marker of B12 status. An increased MMA level is frequently utilized as the gold standard for classifying a patient’s status as B12 deficient or sufficient (32).

However, elevated MMA levels may also be linked with other pathological processes, such as mitochondrial dysfunction and oxidative stress. Mitochondria, the primary sites of energy production in cells, are also the principal sources of oxidative stress. Mitochondrial dysfunction can lead to the accumulation of MMA, even when vitamin B12 levels are sufficient. To delve deeper into the potential link between MMA and mitochondrial dysfunction, it is important to understand the role of MMA in cellular metabolism. MMA is a byproduct of the metabolism of certain amino acids and odd-chain fatty acids. When mitochondrial function is compromised, the ability of cells to metabolize these compounds is hindered, leading to an accumulation of MMA. This accumulation can further exacerbate mitochondrial dysfunction by disrupting the tricarboxylic acid (TCA) cycle, electron transport complex II, and redox homeostasis in mitochondria (35). In addition, several clinical complications, such as chronic kidney disease (36), cardiovascular disease (37), and certain types of cancer (38), are linked with oxidative stress and mitochondrial dysfunction. These diseases may lead to the accumulation of MMA, which explains why MMA levels can be elevated in these patients even when serum B12 levels are within normal or high ranges.

Organs in the body that are rich in mitochondria, such as the heart, kidney, and brain, may be impacted by MMA, which in turn can influence various functions. Numerous studies have shown a close connection between mitochondrial dysfunction and cardiac ischemia–reperfusion injury (39, 40). In addition, improving mitochondrial quality control may mitigate cardiac microvascular ischemia–reperfusion injury (41, 42). The accumulation of circulating MMA promotes an increase of myocardial oxygen consumption, which is related to heart failure and cardiac hypertrophy (20, 43). A retrospective study showed that MMA was elevated in individuals with oxidative stress-related conditions, such as atrial fibrillation or arterial hypertension (37). Research has also illustrated that the level of mitochondria-derived MMA is strongly associated with all-cause and cardiovascular mortality in the general population, particularly those with functional vitamin B12 deficiency (21). Furthermore, MMA has been identified as a risk factor for coronary heart disease in existing cross-sectional studies. In a cross-sectional clinical study encompassing 120 patients, serum MMA levels were significantly elevated in patients suffering from acute myocardial infarction (AMI) or acute heart failure, compared to healthy controls. This was observed to be independent of demographic factors, medical history, or other comorbidities (17). A recent cross-sectional study involving 5,313 participants from the NHANES database showed an independent correlation between elevated serum MMA levels and cardiovascular diseases (CVDs), suggesting that MMA levels could potentially predict the occurrence of CVDs (44). It has also been indicated that the concentration of MMA in patients with chronic decompensated HF is higher than that in newly diagnosed HF patients. This discovery is likely related to the pathophysiology of HF. Chronic HF may lead to mitochondrial dysfunction and oxidative stress, which affects MMA metabolism. Moreover, chronic HF may lead to renal dysfunction, which resulting in reduced clearance of MMA and thus increasing the concentration of MMA in the blood. These findings underscore the importance of monitoring MMA levels in patients with HF, especially those in advanced stages. The increase in MMA levels may not only reflect the status of vitamin B12 deficiency but also the severity of HF and potential mitochondrial dysfunction.

Consistent with previous research, our study found a significant correlation between elevated MMA and HF. However, the novelty of our study lies in the exploration of the impact of MMA on the prognosis of HF patients. A higher MMA levels were closely correlated with increased all-cause mortality in HF population, particularly in those with oxidative risks, including the elderly, smokers, and individuals with hypertension, diabetes and/or overweight. For these HF patients, serum MMA levels could be monitored as a prognostic indicator. However, while these subgroup findings are suggestive, they necessitate careful interpretation due to the exploratory nature of the analyses and inherent limitations associated with the sample sizes of the subgroups.

Our findings also underscore the significant potential of MMA for risk stratification and improved disease management, particularly when functional vitamin B12 deficiency or oxidative stress is present. Nevertheless, the specific clinical thresholds for risk stratification and intervention, notably in relation to cardiovascular disease outcomes, requires further elucidation. In our study, the RCS analysis led to the calculation of an MMA cut-off value of 200.387 nmol/L. It’s important to note, however, that this value, being derived from a single database, should not be considered as a definitive clinical threshold. As previously highlighted, MMA cut-offs reported in the literature exhibit a wide range, spanning from 210 to 480 nmol/L (14). When MMA levels surpass certain clinical threshold, it signals a need to initiate metabolic screening, commence B12 supplementation, and maintain close monitoring of renal function and inflammation markers. Furthermore, regular monitoring of MMA levels can offer valuable insights into the progression of the disease and the efficacy of therapeutic interventions.

Elevated MMA levels could serve a crucial role in risk stratification by helping identify patients at high risk who may benefit from more aggressive management strategies. Moreover, for patients with cardiovascular disease, interventions tailored based on MMA levels, such as personalized vitamin B12 supplementation strategies, could potentially improve patient prognosis. Such precision interventions underscore the potential of MMA in guiding individualized treatment approaches. With the development of emerging technologies like brain-computer interfaces (45), the integration of metabolic biomarkers such as MMA into advanced monitoring systems may offer novel strategies for risk stratification in heart failure management.

This is the first study to explore the association between MMA and all-cause mortality in HF populations. The strengths of our study include the utilization of a large, nationally representative sample, a prospective study design, and the adjustment for several confounding variables using comprehensive data from NHANES. However, several limitations of this study also need to be addressed. Firstly, the inherent limitations of the cross-sectional study design prevent us from establishing causality. Longitudinal studies are needed to validate these findings and explore temporal relationships. Secondly, the relatively smaller number of populations with both HF and MMA data in our study could possibly result in limited statistical power, particularly for stratified analyses where sample sizes were further reduced. Although statistically significant subgroup differences were observed, these results should be interpreted with caution. Sensitivity analyze further highlighted the impact of missing data, suggesting that incomplete datasets may influence the accuracy and robustness of our findings. The exploration of the potential link between MMA and cardiovascular-related mortality is also limited due to the insufficient number of participants and the lack of detailed follow-up data. Thirdly, the identification of heart failure populations was based on self-reporting, which may introduce recall bias and misclassification. Furthermore, the lack of objective clinical data on HF severity, such as NT-proBNP and LVEF measurements, prevented us from assessing potential correlations between MMA levels and the severity of HF. Finally, due to the single measurement of MMA levels, it is impossible to determine the long-term baseline for each individual and to distinguish between innate and postnatal increases of MMA. However, the prevalence of hereditary methylmalonic acidemia in the general population is very low, which should not significantly impact the results.

## Data Availability

The raw data supporting the conclusions of this article will be made available by the authors, without undue reservation.

## Footnotes

^1^ https://www.cdc.gov/nchs/nhanes/index.htm
