# Supplementary material for: Exploring the relationship between vitamin B12, methylmalonic acid levels and all-cause mortality in heart failure populations: insights from the NHANES database
Source: Front Nutr. 2025 Jun 25;12:1597305. doi: 10.3389/fnut.2025.1597305 (PMC12237664; doi:10.3389/fnut.2025.1597305)
Supplement: Supplementary file 1 [file Table_1.docx]

Supplementary Table 1. Comparison of hazard ratios (95% CIs) for all-cause mortality according to serum B12 levels: complete-case analysis vs. Random Forest sensitivity analysis.

| Complete-Case Analysis |  |  | Serum B12, pg/mL | | | |
| --- | --- | --- | --- | --- | --- | --- |
|  | Ln serum B12^*^ | *P* value | Tertile1(<387) | Tertile2(387-606) | Tertile3(>606) | *P* trend^a^ |
| Participants, N(%) | 580(100.0) |  | 195(100.0) | 192(100.0) | 193(100.0) |  |
| Deaths/person-yrs | 411/2621^&^ |  | 137/955 | 138/947 | 139/719 |  |
| Model 2 | 1.17 (0.92-1.50)^#^ | 0.21 | 1(ref.) | 1.37 (0.99-1.89) | 1.38 (0.95-1.98) | 0.08 |
|  |  |  |  |  |  |  |
| Random Forest Sensitivity Analysis |  |  | Serum B12, pg/mL | | | |
|  | Ln serum B12^*^ | *P* value | Tertile1(<448) | Tertile2(448-640) | Tertile3(>640) | *P* trend^a^ |
| Participants, N(%) | 747(100.0) |  | 250(100.0) | 248(100.0) | 249(100.0) |  |
| Deaths/person-yrs | 139/842^&^ |  | 173/1211 | 139/842 | 169/772 |  |
| Model 2 | 1.24(0.98-1.59)^#^ | 0.08 | 1(ref.) | 1.10(0.82-1.51) | 1.54(1.11-2.14) | 0.01 |

Abbreviations: CI, confidence interval; B12, cobalamin (vitamin B12); eGFR, estimated glomerular filtration rate.

*Hazard ratio per 1 unit increases of natural log-transformed serum B12;

& unweighted;

# Values are weighted hazard ratio (95% confidence interval)

a *p* values for trend were assessed using the median level of each tertile serum B12 levels and modeling it as a continuous variable.

Model 2: adjusted for the Age (continuous, year), and Sex (male and female), Race (Other Hispanic, non-Hispanic white, non-Hispanic black, and other race), Smoking status (Never smoking, Former smoker, and Current smoker), Hypertension (Yes and No), Physical activity (Insufficient and Sufficient), and eGFR (continuous, mL/min/1.73 m^2).
