# Supplementary material for: Exploring the relationship between vitamin B12, methylmalonic acid levels and all-cause mortality in heart failure populations: insights from the NHANES database
Source: Front Nutr. 2025 Jun 25;12:1597305. doi: 10.3389/fnut.2025.1597305 (PMC12237664; doi:10.3389/fnut.2025.1597305)
Supplement: Supplementary file 2 [file Table_2.docx]

Supplementary Table 2. Association of the MMA levels with all-cause mortality among participants with heart failure using quartile grouping.

|  | Participants, N | | | | | | |
| --- | --- | --- | --- | --- | --- | --- | --- |
|  |  |  | MMA Levels, nmol/L | | | | |
| All-cause mortality | Ln MMA^*^ | *P* value | Q1(<140) | Q2(140-200) | Q3(200-293) | Q4(>293) | *P* trend^a^ |
| Participants, N(%) | 747(100.0) |  | 191(100.0) | 183(100.0) | 186(100.0) | 187(100.0) |  |
| Deaths/person-yrs | 481/2825^&^ |  | 99/875 | 102/617 | 129/639 | 151/694 |  |
| Crude | 1.92(1.56-2.35)^#^ | <0.01 | 1(ref.) | 1.46 (1.00-2.12) | 2.36 (1.62-3.45) | 3.22 (2.22-4.68) | <0.01 |
| model 1 | 1.56(1.31-1.87) | <0.01 | 1(ref.) | 1.20 (0.87-1.65) | 1.69 (1.19-2.39) | 2.12 (1.53-2.93) | <0.01 |
| model 2 | 1.26 (1.01-1.57) | 0.04 | 1(ref.) | 1.07 (0.74-1.55) | 1.44 (0.98-2.11) | 1.46 (1.01-2.13) | 0.02 |
| model 3 | 1.37 (1.11-1.68) | <0.01 | 1(ref.) | 1.18 (0.82-1.70) | 1.56 (1.03-2.36) | / | 0.08 |
| model 4 | 1.24 (0.97-1.59) | 0.08 | 1(ref.) | 1.08 (0.73-1.59) | 1.35 (0.88-2.05) | 1.36 (0.91-2.03) | 0.09 |

Abbreviations: MMA, methylmalonic acid; eGFR, estimated glomerular filtration rate; B12, cobalamin (vitamin B12).

*Hazard ratio per 1 unit increases of natural log-transformed MMA.

& unweighted.

# Values are weighted hazard ratio (95% confidence interval).

a *p* values for trend were assessed using the median level of each tertile MMA levels and modeling it as a continuous variable.

Model 1: adjusted for the Age (continuous, year), and Sex (male and female), and Race (Other Hispanic, non-Hispanic white, non-Hispanic black, and other race).

Model 2: adjusted for the Age (continuous, year), and Sex (male and female), Race (Other Hispanic, non-Hispanic white, non-Hispanic black, and other race), Smoking status (Never smoking, Former smoker, and Current smoker), Hypertension (Yes and No), Physical activity (Insufficient and Sufficient), and eGFR (continuous, mL/min/1.73 m^2).

Model 3: further adjustment for vitamin B12 intake from diet (continuous, ug/d) based on Model 2.

Model 4: further adjustment for serum vitamin B12 (continuous, pg/mL) based on Model 2.
